# A Cuckoo Search Algorithm for Multimodal Optimization

**DOI:** 10.1155/2014/497514

**Published:** 2014-07-22

**Authors:** Erik Cuevas, Adolfo Reyna-Orta

**Affiliations:** Departamento de Electronica, Universidad de Guadalajara, CUCEI, Avenida Revolución 1500, 44430 Guadalajara, JAL, Mexico

## Abstract

Interest in multimodal optimization is expanding rapidly, since many practical engineering problems demand the localization of multiple optima within a search space. On the other hand, the cuckoo search (CS) algorithm is a simple and effective global optimization algorithm which can not be directly applied to solve multimodal optimization problems. This paper proposes a new multimodal optimization algorithm called the multimodal cuckoo search (MCS). Under MCS, the original CS is enhanced with multimodal capacities by means of (1) the incorporation of a memory mechanism to efficiently register potential local optima according to their fitness value and the distance to other potential solutions, (2) the modification of the original CS individual selection strategy to accelerate the detection process of new local minima, and (3) the inclusion of a depuration procedure to cyclically eliminate duplicated memory elements. The performance of the proposed approach is compared to several state-of-the-art multimodal optimization algorithms considering a benchmark suite of fourteen multimodal problems. Experimental results indicate that the proposed strategy is capable of providing better and even a more consistent performance over existing well-known multimodal algorithms for the majority of test problems yet avoiding any serious computational deterioration.

## 1. Introduction

Optimization is a field with applications in many areas of science, engineering, economics, and others, where mathematical modelling is used [1]. In general, the goal is to find an acceptable solution of an objective function defined over a given search space. Optimization algorithms are usually broadly divided into deterministic and stochastic ones [2]. Since deterministic methods only provide a theoretical guarantee of locating a local minimum for the objective function, they often face great difficulties in solving optimization problems [3]. On the other hand, stochastic methods are usually faster in locating a global optimum [4]. Moreover, they adapt easily to black-box formulations and extremely ill-behaved functions, whereas deterministic methods usually rest on at least some theoretical assumptions about the problem formulation and its analytical properties (such as Lipschitz continuity) [5].

Evolutionary algorithms (EA), which are considered to be members of the stochastic group, have been developed by a combination of rules and randomness that mimics several natural phenomena. Such phenomena include evolutionary processes such as the evolutionary algorithm (EA) proposed by Fogel et al. [6], de Jong [7], and Koza [8]; the genetic algorithm (GA) proposed by Holland [9] and Goldberg [10]; the artificial immune system proposed by de Castro and von Zuben [11]; and the differential evolution algorithm (DE) proposed by Storn and Price [12]. Some other methods which are based on physical processes include simulated annealing proposed by Kirkpatrick et al. [13], the electromagnetism-like algorithm proposed by Birbil and Fang [14], and the gravitational search algorithm proposed by Rashedi et al. [15]. Also, there are other methods based on the animal-behavior phenomena such as the particle swarm optimization (PSO) algorithm proposed by Kennedy and Eberhart [16] and the ant colony optimization (ACO) algorithm proposed by Dorigo et al. [17].

Most of research work on EA aims for locating the global optimum [18]. Despite its best performance, a global optimum may be integrated by parameter values that are considered impractical or prohibitively expensive, limiting their adoption into a real-world application. Therefore, from a practical point of view, it is desirable to have access to not only the global optimum but also as many local optima as possible (ideally all of them). Under such circumstances, a local optimum with acceptable performance quality and modest cost may be preferred over a costly global solution with marginally better performance [19]. The process of finding the global optimum and multiple local optima is known as multimodal optimization.

EA perform well for locating a single optimum but fail to provide multiple solutions [18]. Several methods have been introduced into the EA scheme to achieve multimodal optimization, such as fitness sharing [20–22], deterministic crowding [23], probabilistic crowding [22, 24], clustering based niching [25], clearing procedure [26], species conserving genetic algorithm [27], and elitist-population strategies [28]. However, most of these methods have difficulties that need to be overcome before they can be employed successfully to multimodal applications. Some identified problems include difficulties in tuning some niching parameters, difficulties in maintaining discovered solutions in a run, extra computational overheads, and poor scalability when dimensionality is high. An additional problem represents the fact that such methods are devised for extending the search capacities of popular EA such as GA and PSO, which fail in finding a balance between exploration and exploitation, mainly for multimodal functions [29]. Furthermore, they do not explore the whole region effectively and often suffer premature convergence or loss of diversity.

As alternative approaches, other researchers have employed artificial immune systems (AIS) to solve multimodal optimization problems. Some examples are the clonal selection algorithm [30] and the artificial immune network (AiNet) [31, 32]. Both approaches use operators and structures which attempt to find multiple solutions by mimicking the natural immune system's behavior.

Every EA needs to address the issue of exploration and exploitation of the search space [33]. Exploration is the process of visiting entirely new points of a search space, whilst exploitation is the process of refining those points within the neighborhood of previously visited locations in order to improve their solution quality. Pure exploration degrades the precision of the evolutionary process but increases its capacity to find new potential solutions. On the other hand, pure exploitation allows refining existent solutions but adversely driving the process to local optimal solutions.

Multimodal optimization requires a sufficient amount of exploration of the population agents in hyperspace so that all the local and global attractors can be successfully and quickly detected [34, 35]. However, an efficient multimodal optimization algorithm should exhibit not only good exploration tendency but also good exploitative power, especially during the last stages of the search, because it must ensure accurately a distributed convergence to different optima in the landscape. Therefore, the ability of an EA to find multiple solutions depends on its capacity to reach a good balance between the exploitation of found-so-far elements and the exploration of the search space [36]. So far, the exploration-exploitation dilemma has been an unsolved issue within the framework of EA.

Recently, a novel nature-inspired algorithm, called the cuckoo search (CS) algorithm [37], has been proposed for solving complex optimization problems. The CS algorithm is based on the obligate brood-parasitic strategy of some cuckoo species. One of the most powerful features of CS is the use of Lévy flights to generate new candidate solutions. Under this approach, candidate solutions are modified by employing many small changes and occasionally large jumps. As a result, CS can substantially improve the relationship between exploration and exploitation, still enhancing its search capabilities [38]. Recent studies show that CS is potentially far more efficient than PSO and GA [39]. Such characteristics have motivated the use of CS to solve different sorts of engineering problems such as mesh generation [40], embedded systems [41], steel frame design [42], scheduling problems [43], thermodynamics [44], and distribution networks [45].

This paper presents a new multimodal optimization algorithm called the multimodal cuckoo search (MCS). The method combines the CS algorithm with a new memory mechanism which allows an efficient registering of potential local optima according to their fitness value and the distance to other potential solutions. The original CS selection strategy is mainly conducted by an elitist decision where the best individuals prevail. In order to accelerate the detection process of potential local minima in our method, the selection strategy is modified to be influenced by individuals that are contained in the memory mechanism. During each generation, eggs (individuals) that exhibit different positions are included in the memory. Since such individuals could represent to the same local optimum, a depuration procedure is also incorporated to cyclically eliminate duplicated memory elements. The performance of the proposed approach is compared to several state-of-the-art multimodal optimization algorithms considering a benchmark suite of 14 multimodal problems. Experimental results indicate that the proposed strategy is capable of providing better and even more consistent performance over the existing well-known multimodal algorithms for the majority of test problems avoiding any serious computational deterioration.

The paper is organized as follows.  Section 2 explains the cuckoo search (CS) algorithm, while Section 3 presents the proposed MCS approach.  Section 4 exhibits the experimental set and its performance results. Finally, Section 5 establishes some concluding remarks.

## 2. Cuckoo Search (CS) Method

CS is one of the latest nature-inspired algorithms, developed by Yang and Deb [37]. CS is based on the brood parasitism of some cuckoo species. In addition, this algorithm is enhanced by the so-called Lévy flights [46], rather than by simple isotropic random walks. Recent studies show that CS is potentially far more efficient than PSO and GA [39].

Cuckoo birds lay their eggs in the nests of other host birds (usually other species) with amazing abilities such as selecting nests containing recently laid eggs and removing existing eggs to increase the hatching probability of their own eggs. Some of the host birds are able to combat this parasitic behavior of cuckoos and throw out the discovered alien eggs or build a new nest in a distinct location. This cuckoo breeding analogy is used to develop the CS algorithm. Natural systems are complex, and therefore they cannot be modeled exactly by a computer algorithm in its basic form. Simplification of natural systems is necessary for successful implementation in computer algorithms. Yang and Deb [39] simplified the cuckoo reproduction process into three idealized rules.

1. An egg represents a solution and is stored in a nest. An artificial cuckoo can lay only one egg at a time.
2. The cuckoo bird searches for the most suitable nest to lay the eggs in (solution) to maximize its eggs' survival rate. An elitist selection strategy is applied, so that only high-quality eggs (best solutions near the optimal value) which are more similar to the host bird's eggs have the opportunity to develop (next generation) and become mature cuckoos.
3. The number of host nests (population) is fixed. The host bird can discover the alien egg (worse solutions away from the optimal value) with a probability of *p*_*a*_ ∈ [0,1], and these eggs are thrown away or the nest is abandoned and a completely new nest is built in a new location. Otherwise, the egg matures and lives to the next generation. New eggs (solutions) laid by a cuckoo choose the nest by Lévy flights around the current best solutions.

From the implementation point of view, in the CS operation, a population, **E** ^*k*^ ({**e**_1_^*k*^, **e**_2_^*k*^,…, **e**_*N*_^*k*^}), of *N* eggs (individuals) is evolved from the initial point (*k* = 0) to a total gen number iterations (*k* = 2 · gen). Each egg, **e**_*i*_^*k*^ (*i* ∈ [1,…, *N*]), represents an *n*-dimensional vector, {*e*_*i*,1_^*k*^, *e*_*i*,2_^*k*^,…, *e*_*i*,*n*_^*k*^}, where each dimension corresponds to a decision variable of the optimization problem to be solved. The quality of each egg, **e**_*i*_^*k*^ (candidate solution), is evaluated by using an objective function, *f*(**e**_*i*_^*k*^), whose final result represents the fitness value of **e**_*i*_^*k*^.Three different operators define the evolution process of CS: (A) Lévy flight, (B) replacement of some nests by constructing new solutions, and (C) elitist selection strategy.

### 2.1. Lévy Flight (A)

One of the most powerful features of cuckoo search is the use of Lévy flights to generate new candidate solutions (eggs). Under this approach, a new candidate solution, **e**_*i*_^*k*+1^ (*i* ∈ [1,…, *N*]), is produced by perturbing the current **e**_*i*_^*k*^ with a change of position **c**_*i*_. In order to obtain **c**_*i*_, a random step, **s**_*i*_, is generated by a symmetric Lévy distribution. For producing **s**_*i*_, Mantegna's algorithm [47] is employed as follows:

(1) $$\begin{array}{c} s_{i}=\frac{u}{{\left|v\right|}^{1/\beta }}, \end{array}$$

where **u**({*u*_1_,…, *u*_*n*_}) and **v**  ({*v*_1_,…, *v*_*n*_}) are *n*-dimensional vectors and *β* = 3/2. Each element of **u** and **v** is calculated by considering the following normal distributions:

(2) $$\begin{array}{c} u\sim N\left(0,\sigma_{u}^{2}\right),\;\;v\sim N\left(0,\sigma_{v}^{2}\right),\\ \sigma_{u}={\left(\frac{\Gamma \left(1+\beta \right)\cdot \sin\left(\pi \cdot \beta /2\right)}{\Gamma \left(\left(1+\beta \right)/2\right)\cdot \beta \cdot 2^{(\beta -1)/2}}\right)}^{1/\beta },\;\;\sigma_{v}=1, \end{array}$$

where Γ(·) represents the gamma distribution. Once **s**_*i*_ has been calculated, the required change of position **c**_*i*_ is computed as follows:

(3) $$\begin{array}{c} c_{i}=0.01\cdot s_{i}⊕\left(e_{i}^{k}-e^{\text{best}}\right), \end{array}$$

where the product ⊕ denotes entrywise multiplications whereas **e** ^best^ is the best solution (egg) seen so far in terms of its fitness value. Finally, the new candidate solution, **e**_*i*_^*k*+1^, is calculated by using

(4) $$\begin{array}{c} e_{i}^{k+1}=e_{i}^{k}+c_{i}. \end{array}$$

### 2.2. Replacement of Some Nests by Constructing New Solutions (B)

Under this operation, a set of individuals (eggs) are probabilistically selected and replaced with a new value. Each individual, **e**_*i*_^*k*^ (*i* ∈ [1,…, *N*]), can be selected with a probability of *p*_*a*_ ∈ [0,1]. In order to implement this operation, a uniform random number, *r*_1_, is generated within the range [0, 1]. If *r*_1_ is less than *p*_*a*_, the individual **e**_*i*_^*k*^ is selected and modified according to (5). Otherwise, **e**_*i*_^*k*^ remains without change. This operation can be resumed by the following model:

(5) $$\begin{array}{c} e_{i}^{k+1}=\{\begin{array}{cc} e_{i}^{k}+\text{rand}\cdot \left(e_{d_{1}}^{k}-e_{d_{2}}^{k}\right), & \text{with}\;\text{probability}\;\;p_{a},\\ e_{i}^{k}, & \text{with}\;\text{probability}\;\left(1-p_{a}\right), \end{array} \end{array}$$

where rand is a random number normally distributed, whereas *d*_1_ and *d*_2_ are random integers from 1 to *N*.

### 2.3. Elitist Selection Strategy (C)

After producing **e**_*i*_^*k*+1^ either by operator A or by operator B, it must be compared with its past value **e**_*i*_^*k*^. If the fitness value of **e**_*i*_^*k*+1^ is better than **e**_*i*_^*k*^, then **e**_*i*_^*k*+1^ is accepted as the final solution. Otherwise, **e**_*i*_^*k*^ is retained. This procedure can be resumed by the following statement:

(6) $$\begin{array}{c} e_{i}^{k+1}=\{\begin{array}{cc} e_{i}^{k+1}, & \text{if}\;\;f\left(e_{i}^{k+1}\right)<f\left(e_{i}^{k}\right),\\ e_{i}^{k}, & \text{otherwise}. \end{array} \end{array}$$

This elitist selection strategy denotes that only high-quality eggs (best solutions near the optimal value) which are more similar to the host bird's eggs have the opportunity to develop (next generation) and become mature cuckoos.

### 2.4. Complete CS Algorithm

CS is a relatively simple algorithm with only three adjustable parameters: *p*_*a*_, the population size, *N*, and the number of generations gen. According to Yang and Deb [39], the convergence rate of the algorithm is not strongly affected by the value of *p*_*a*_ and it is suggested to use *p*_*a*_ = 0.25. The operation of CS is divided in two parts: initialization and the evolution process. In the initialization (*k* = 0), the first population, **E** ^0^ ({**e**_1_^0^, **e**_2_^0^,…, **e**_*N*_^0^}), is produced. The values, {*e*_*i*,1_^0^, *e*_*i*,2_^0^,…, *e*_*i*,*n*_^0^}, of each individual, **e**_*i*_^*k*^, are randomly and uniformly distributed between the prespecified lower initial parameter bound, *b*_*j*_^low^, and the upper initial parameter bound, *b*_*j*_^high^. One has

(7) ei,j0=bjlow+rand·(bjhigh−bjlow);i=1,2,…,N; j=1,2,…,n.

In the evolution process, operators A (Lévy flight), B (replacement of some nests by constructing new solutions), and C (elitist selection strategy) are iteratively applied until the number of iterations *k* = 2 · gen has been reached. The complete CS procedure is illustrated in Algorithm 1.

From Algorithm 1, it is important to remark that the elitist selection strategy (C) is used two times, just after operator A or operator B is executed.

## 3. The Multimodal Cuckoo Search (MCS)

In CS, individuals emulate eggs which interact in a biological system by using evolutionary operations based on the breeding behavior of some cuckoo species. One of the most powerful features of CS is the use of Lévy flights to generate new candidate solutions. Under this approach, candidate solutions are modified by employing many small changes and occasionally large jumps. As a result, CS can substantially improve the relationship between exploration and exploitation, still enhancing its search capabilities. Despite such characteristics, the CS method still fails to provide multiple solutions in a single execution. In the proposed MCS approach, the original CS is adapted to include multimodal capacities. In particular, this adaptation contemplates (1) the incorporation of a memory mechanism to efficiently register potential local optima according to their fitness value and the distance to other potential solutions, (2) the modification of the original CS individual selection strategy to accelerate the detection process of new local minima, and (3) the inclusion of a depuration procedure to cyclically eliminate duplicated memory elements.

**Figure 1:**
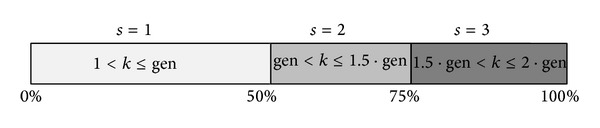
Division of the evolution process according to MCS.

In order to implement these modifications, the proposed MCS divides the evolution process in three asymmetric states. The first state (*s* = 1) includes 0 to 50% of the evolution process. The second state (*s* = 2) involves 50 to 75%. Finally, the third state (*s* = 3) lasts from 75 to 100%. The idea of this division is that the algorithm can react in a different manner depending on the current state. Therefore, in the beginning of the evolutionary process, exploration can be privileged, while, at the end of the optimization process, exploitation can be favored.  Figure 1 illustrates the division of the evolution process according to MCS.

The next sections examine the operators suggested by MCS as adaptations of CS to provide multimodal capacities. These operators are (D) the memory mechanism, (E) new selection strategy, and (F) depuration procedure.

### 3.1. Memory Mechanism (D)

In the MCS evolution process, a population, **E** ^*k*^ ({**e**_1_^*k*^, **e**_2_^*k*^,…, **e**_*N*_^*k*^}), of *N* eggs (individuals) is evolved from the initial point (*k* = 0) to a total gen number iterations (*k* = 2 · gen). Each egg, **e**_*i*_^*k*^ (*i* ∈ [1,…, *N*]), represents an *n*-dimensional vector, {*e*_*i*,1_^*k*^, *e*_*i*,2_^*k*^,…, *e*_*i*,*n*_^*k*^}, where each dimension corresponds to a decision variable of the optimization problem to be solved. The quality of each egg, **e**_*i*_^*k*^ (candidate solution), is evaluated by using an objective function, *f*(**e**_*i*_^*k*^), whose final result represents the fitness value of **e**_*i*_^*k*^. During the evolution process, MCS maintains also the best, **e** ^best,*k*^, and the worst, **e** ^worst,*k*^, eggs seen so far, such that

(8) $$\begin{array}{c} e^{\text{best},k}=\underset{i\in \left\{1,2,\ldots ,N\right\},a\in \left\{1,2,\ldots ,k\right\}}{\arg\;\min}\left(f\left(e_{i}^{a}\right)\right),\\ e^{\text{worst},k}=\underset{i\in \left\{1,2,\ldots ,N\right\},a\in \left\{1,2,\ldots ,k\right\}}{\arg\;\min}\left(f\left(e_{i}^{a}\right)\right). \end{array}$$

Global and local optima possess two important characteristics: (1) they have a significant good fitness value and (2) they represent the best fitness value inside a determined neighborhood. Therefore, the memory mechanism allows efficiently registering potential global and local optima during the evolution process, involving a memory array, **M**, and a storage procedure. **M** stores the potential global and local optima, {**m**_1_, **m**_2_,…, **m**_*T*_}, during the evolution process, with *T* being the number of elements so far that are contained in the memory **M**. On the other hand, the storage procedure indicates the rules that the eggs, {**e**_1_^*k*^, **e**_2_^*k*^,…, **e**_*N*_^*k*^}, must fulfill in order to be captured as memory elements. The memory mechanism operates in two phases: initialization and capture.

#### 3.1.1. Initialization Phase

This phase is applied only once within the optimization process. Such an operation is achieved in the null iteration (*k* = 0) of the evolution process. In the initialization phase, the best egg, **e**_*B*_, of **E** ^0^, in terms of its fitness value, is stored in the memory** M** (**m**_1_ = **e**_*B*_), where **e**_*B*_ = arg min⁡_*i*∈{1,2,…,*N*}_⁡(*f*(**e**_*i*_^0^)), for a minimization problem.

#### 3.1.2. Capture Phase

This phase is applied from the first (*k* = 1) iteration to the last iteration (*k* = 2,3,…, 2 · gen), at the end of each operator (A and B). At this stage, eggs, {**e**_1_^*k*^, **e**_2_^*k*^,…, **e**_*N*_^*k*^}, corresponding to potential global and local optima are efficiently registered as memory elements, {**m**_1_, **m**_2_,…, **m**_*T*_}, according to their fitness value and the distance to other potential solutions. In the operation, each egg, **e**_*i*_^*k*^, of **E** ^*k*^ is tested in order to evaluate if it must be captured as a memory element. The test considers two rules: (1) significant fitness value rule and (2) nonsignificant fitness value rule.

*Significant Fitness Value Rule*. Under this rule, the solution quality of **e**_*i*_^*k*^ is evaluated according to the worst element, **m** ^worst^, that is contained in the memory** M**, where **m** ^worst^ = arg max⁡_*i*∈{1,2,…,*T*}_⁡(*f*(**m**_*i*_)), in case of a minimization problem. If the fitness value of **e**_*i*_^*k*^ is better than **m** ^worst^(*f*(**e**_*i*_^*k*^) < *f*(**m** ^worst^)), **e**_*i*_^*k*^ is considered potential global and local optima. The next step is to decide whether **e**_*i*_^*k*^ represents a new optimum or it is very similar to an existent memory element, {**m**_1_, **m**_2_,…, **m**_*T*_} (if it is already contained in the memory** M**). Such a decision is specified by an acceptance probability function, Pr(*δ*_*i*,*u*_, *s*), that depends, on one side, on the distances *δ*_*i*,*u*_ from **e**_*i*_^*k*^ to the nearest memory element **m**_*u*_ and, on the other side, on the current state *s* of the evolution process (1, 2, and 3). Under Pr(*δ*_*i*,*u*_, *s*), the probability that **e**_*i*_^*k*^ would be part of** M** increases as the distance *δ*_*i*,*u*_ enlarges. Similarly, the probability that **e**_*i*_^*k*^ would be similar to an existent memory element {**m**_1_, **m**_2_,…, **m**_*T*_} increases as *δ*_*i*,*u*_ decreases. On the other hand, the indicator *s* that relates a numeric value with the state of the evolution process is gradually modified during the algorithm to reduce the likelihood of accepting inferior solutions. The idea is that in the beginning of the evolutionary process (exploration), large distance differences can be considered, while only small distance differences are tolerated at the end of the optimization process.

In order to implement this procedure, the normalized distance *δ*_*i*,*q*_ (*q* ∈ [1,…, *T*]) is calculated from **e**_*i*_^*k*^ to all the elements of the memory** M**  {**m**_1_, **m**_2_,…, **m**_*T*_}. *δ*_*i*,*q*_ is computed as follows:

(9) $$\begin{array}{c} \delta_{i,q}=\sqrt{{\left(\frac{e_{i,1}^{k}-m_{q,1}}{b_{1}^{\text{high}}-b_{1}^{\text{low}}}\right)}^{2}+{\left(\frac{e_{i,2}^{k}-m_{q,2}}{b_{2}^{\text{high}}-b_{2}^{\text{low}}}\right)}^{2}+\cdots +{\left(\frac{e_{i,n}^{k}-m_{q,n}}{b_{n}^{\text{high}}-b_{n}^{\text{low}}}\right)}^{2}}, \end{array}$$

where {*m*_*q*,1_, *m*_*q*,2_,…, *m*_*q*,*n*_} represent the *n* components of the memory element **m**_*q*_, whereas *b*_*j*_^high^ and *b*_*j*_^low^ indicate the low *j* parameter bound and the upper *j* parameter bound (*j* ∈ [1,…, *n*]), respectively. One important property of the normalized distance *δ*_*i*,*q*_ is that its values fall into the interval [0,1].

By using the normalized distance *δ*_*i*,*q*_ the nearest memory element **m**_*u*_ to **e**_*i*_^*k*^ is defined, with **m**_*u*_ = arg min⁡_*j*∈{1,2,…,*T*}_⁡(*δ*_*i*,*j*_). Then, the acceptance probability function Pr(*δ*_*i*,*u*_, *s*) is calculated by using the following expression:

(10) $$\begin{array}{c} \text{Pr}\left(\delta_{i,u},s\right)={\left(\delta_{i,u}\right)}^{s}. \end{array}$$

In order to decide whether **e**_*i*_^*k*^ represents a new optimum or it is very similar to an existent memory element, a uniform random number *r*_1_ is generated within the range [0, 1]. If *r*_1_ is less than Pr(*δ*_*i*,*u*_, *s*), the egg **e**_*i*_^*k*^ is included in the memory** M **as a new optimum. Otherwise, it is considered that **e**_*i*_^*k*^ is similar to **m**_*u*_. Under such circumstances, the memory** M** is updated by the competition between **e**_*i*_^*k*^ and **m**_*u*_, according to their corresponding fitness values. Therefore, **e**_*i*_^*k*^ would replace **m**_*u*_ in case *f*(**e**_*i*_^*k*^) is better than *f*(**m**_*u*_). On the other hand, if *f*(**m**_*u*_) is better than *f*(**e**_*i*_^*k*^), **m**_*u*_ remains with no change. The complete procedure of the significant fitness value rule can be resumed by the following statement:

(11) M={mT+1=eik, with  probability  Pr(δi,u,s),mu=eik if  f(eik)<f(mu), with  probability  1−Pr(δi,u,s).

**Figure 2:**
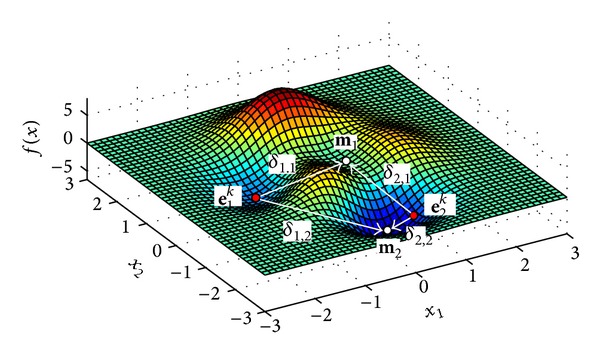
Graphical illustration of the significant fitness value rule process.

In order to demonstrate the significant fitness value rule process, Figure 2 illustrates a simple minimization problem that involves a two-dimensional function, *f*(**x**)  (**x** = {*x*_1_, *x*_2_}). As an example, it assumed a population, **E** ^*k*^, of two different particles (**e**_1_^*k*^, **e**_2_^*k*^), a memory with two memory elements (**m**_1_, **m**_2_), and the execution of the first state (*s* = 1). According to Figure 2, both particles **e**_1_^*k*^ and **e**_2_^*k*^ maintain a better fitness value than **m**_1_, which possesses the worst fitness value of the memory elements. Under such conditions, the significant fitness value rule must be applied to both particles. In case of **e**_1_^*k*^, the first step is to calculate the correspondent distances *δ*_1,1_ and *δ*_1,2_. **m**_1_ represents the nearest memory element to **e**_1_^*k*^. Then, the acceptance probability function Pr(*δ*_1,1_, 1) is calculated by using (10). Since the value of Pr(*δ*_1,1_, 1) is high, there exists a great probability that **e**_1_^*k*^ becomes the next memory element (**m**_3_ = **e**_1_^*k*^). On the other hand, for **e**_2_^*k*^, **m**_2_ represents the nearest memory element. As Pr(*δ*_2,2_, 1) is very low, there exists a great probability that **e**_2_^*k*^ competes with **m**_2_ for a place within** M**. In such a case, **m**_2_ remains with no change considering that *f*(**m**_2_) < *f*(**e**_2_^*k*^).

*Nonsignificant Fitness Value Rule*. Different to the significant fitness value rule, the nonsignificant fitness value rule allows capturing local optima with low fitness values. It operates if the fitness value of **e**_*i*_^*k*^ is worse than **m** ^worst^ (*f*(**e**_*i*_^*k*^) ≥ *f*(**m** ^worst^)). Under such conditions, it is necessary, as a first step, to test which particles could represent local optima and which must be ignored as a consequence of their very low fitness value. Then, if the particle represents a possible local optimum, its inclusion inside the memory** M** is explored.

**Figure 3:**
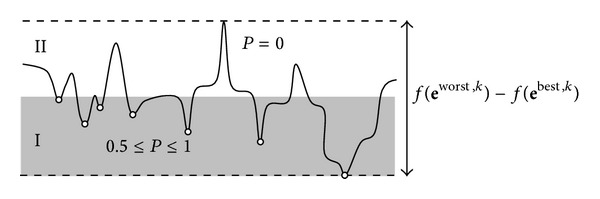
Effect of the probability function *P* in a simple example.

The decision on whether **e**_*i*_^*k*^ represents a new local optimum or not is specified by a probability function, *P*, which is based on the relationship between *f*(**e**_*i*_^*k*^) and the so far valid fitness value interval (*f*(**e** ^worst,*k*^) − *f*(**e** ^best,*k*^)). Therefore, the probability function *P* is defined as follows:

(12) $$\begin{array}{c} p\left(e_{i}^{k},e^{\text{best},k},e^{\text{worst},k}\right)=1-\frac{f\left(e_{i}^{k}\right)-f\left(e^{\text{best},k}\right)}{f\left(e^{\text{wors}t,k}\right)-f\left(e^{\text{best},k}\right)},\\ P\left(p\right)=\{\begin{array}{cc} p, & 0.5\leq p\leq 1,\\ 0, & 0\leq p<0.5, \end{array} \end{array}$$

where **e** ^best,*k*^ and **e** ^worst,*k*^ represent the best and worst eggs seen so far, respectively. In order to decide whether **p**_*i*_^*k*^ represents a new local optimum or it must be ignored, a uniform random number, *r*_2_, is generated within the range [0, 1]. If *r*_2_ is less than *P*, the egg **e**_*i*_^*k*^ is considered to be a new local optimum. Otherwise, it must be ignored. Under *P*, the so far valid fitness value interval (*f*(**e** ^worst,*k*^) − *f*(**e** ^best,*k*^)) is divided into two sections: I and II (see Figure 3). Considering this division, the function *P* assigns a valid probability (greater than zero) only to those eggs that fall into the zone of the best individuals (part I) in terms of their fitness value. Such a probability value increases as the fitness value improves. The complete procedure can be reviewed in Algorithm 2.

If the particle represents a possible local optimum, its inclusion inside the memory** M **is explored. In order to consider if **e**_*i*_^*k*^ could represent a new memory element, another procedure that is similar to the significant fitness value rule process is applied. Therefore, the normalized distance *δ*_*i*,*q*_ (*q* ∈ [1,…, *T*]) is calculated from **p**_*i*_^*k*^ to all the elements of the memory** M **{**m**_1_, **m**_2_,…, **m**_*T*_}, according to (9). Afterwards, the nearest distance *δ*_*i*,*u*_ to **e**_*i*_^*k*^ is determined. Then, by using Pr(*δ*_*i*,*u*_, *s*) (10), the following rule can be thus applied:

(13) $$\begin{array}{c} M=\{\begin{array}{cc} m_{T+1}=e_{i}^{k}, & \text{with}\;\text{probability}\;\text{Pr}\left(\delta_{i,u},s\right),\\ \text{no}\;\text{change}, & \text{with}\;\text{probability}\;1-\text{Pr}\left(\delta_{i,u},s\right). \end{array} \end{array}$$

Under this rule, a uniform random number, *r*_3_, is generated within the range [0, 1]. If *r*_3_ is less than Pr(*δ*_*i*,*u*_, *s*), the egg **e**_*i*_^*k*^ is included in the memory** M** as a new optimum. Otherwise, the memory does not change.

### 3.2. New Selection Strategy (E)

The original CS selection strategy is mainly conducted by an elitist decision where the best individuals in the current population prevail. Such an operation, defined in this paper as operator C (Section 2.3), is executed two times, just after operators A and B in the original CS method. This effect allows incorporating interesting convergence properties to CS when the objective considers only one optimum. However, in case of multiple-optimum detection, such a strategy is not appropriate. Therefore, in order to accelerate the detection process of potential local minima in our method, the selection strategy is modified to be influenced by the individuals contained in the memory** M**.

Under the new selection procedure (operator E), the final population **E** ^*k*+1^ is built by considering the first *N* element from the memory** M** instead of using the best individuals between the currents **E** ^*k*+1^ and **E** ^*k*^. In case of the number of elements in** M** is less than *N*, the rest of the individuals are completed by considering the best elements from the current **E** ^*k*+1^.

### 3.3. Depuration Procedure (F)

During the evolution process, the memory** M** stores several individuals (eggs). Since such individuals could represent the same local optimum, a depuration procedure is incorporated at the end of each state *s* (1, 2, and 3) to eliminate similar memory elements. The inclusion of this procedure allows (a) reducing the computational overhead during each state and (b) improving the search strategy by considering only significant memory elements.

Memory elements tend to concentrate on optimal points (good fitness values), whereas element concentrations are enclosed by areas holding bad fitness values. The main idea in the depuration procedure is to find the distances among concentrations. Such distances, considered as depuration ratios, are later employed to delete all elements inside them, except for the best element in terms of their fitness values.

The method used by the depuration procedure in order to determine the distance between two concentrations is based on the element comparison between the concentration corresponding to the best element and the concentration of the nearest optimum in the memory. In the process, the best element **m** ^best^ in the memory is compared to a memory element, **m**_*b*_, which belongs to one of both concentrations (where **m** ^best^ = arg min⁡_*i*∈{1,2,…,*T*}_⁡(*f*(**m**_*i*_))). If the fitness value of the medium point, *f*((**m** ^best^ + **m**_*b*_)/2), between both is not worse than both, (*f*(**m** ^best^), *f*(**m**_*b*_)), the element **m**_*b*_ is part of the same concentration of **m** ^best^. However, if *f*((**m** ^best^ + **m**_*b*_)/2) is worse than both, the element **m**_*b*_ is considered as part of the nearest concentration. Therefore, if **m**_*b*_ and **m** ^best^ belong to different concentrations, the Euclidian distance between **m**_*b*_ and **m** ^best^ can be considered as a depuration ratio. In order to avoid the unintentional deletion of elements in the nearest concentration, the depuration ratio *D*_*R*_ is lightly shortened. Thus, the depuration ratio *r* is defined as follows:

(14) $$\begin{array}{c} D_{R}=0.85\cdot ||m^{\text{best}}-m_{b}||. \end{array}$$

The proposed depuration procedure only considers the depuration ratio *r* between the concentration of the best element and the nearest concentration. In order to determine all ratios, preprocessing and postprocessing methods must be incorporated and iteratively executed.

The preprocessing method must (1) obtain the best element **m** ^best^ from the memory in terms of its fitness value, (2) calculate the Euclidian distances from the best element to the rest of the elements in the memory, and (3) sort the distances according to their magnitude. This set of tasks allows identification of both concentrations: the one belonging to the best element and that belonging to the nearest optimum, so they must be executed before the depuration ratio *D*_*R*_ calculation. Such concentrations are represented by the elements with the shortest distances to **m** ^best^. Once *D*_*R*_ has been calculated, it is necessary to remove all the elements belonging to the concentration of the best element. This task is executed as a postprocessing method in order to configure the memory for the next step. Therefore, the complete depuration procedure can be represented as an iterative process that at each step determines the distance of the concentration of the best element with regard to the concentration of the nearest optimum.

A special case can be considered when only one concentration is contained within the memory. This case can happen because the optimization problem has only one optimum or because all the other concentrations have been already detected. Under such circumstances, the condition where *f*((**m** ^best^ + **m**_*b*_)/2) is worse than *f*(**m** ^best^) and *f*(**m**_*b*_) would be never fulfilled.

In order to find the distances among concentrations, the depuration procedure is conducted in Procedure 1.

At the end of the above procedure, the vector **Y** will contain the depurated memory which would be used in the next state or as a final result of the multimodal problem.

**Figure 4:**
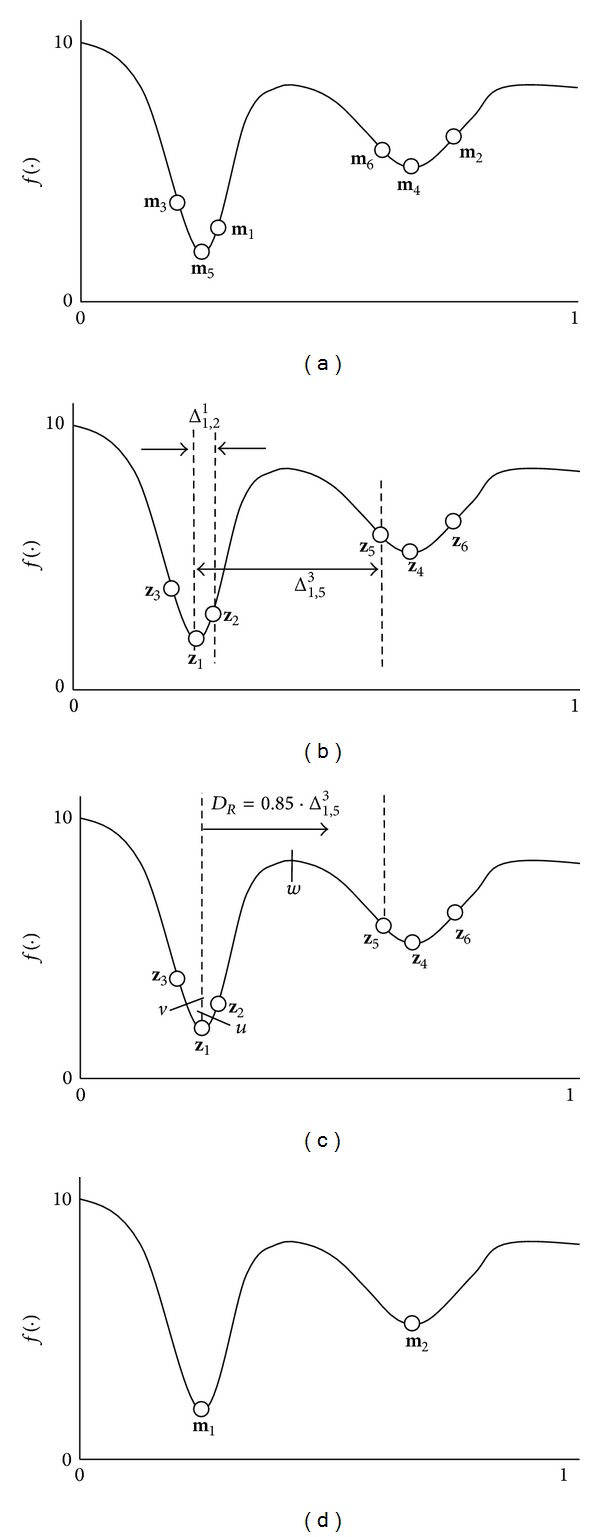
Depuration procedure. (a) Initial memory configuration, (b) vector** Z** and distances Δ_1,*j*_^*a*^, (c) the determination of the depuration ratio *R*, and (d) the final memory configuration.

In order to illustrate the depuration procedure, Figure 4 shows a simple minimization problem that involves two different optimal points (concentrations). As an example, it assumed a memory, **M**, with six memory elements whose positions are shown in Figure 4(a). According to the depuration procedure, the first step is (1) to build the vector **Z** and (2) to calculate the corresponding distance Δ_1,*j*_^*a*^ among the elements. Following such operation, the vector** Z **and the set of distances are configured as **Z** = {**m**_5_, **m**_1_, **m**_3_, **m**_4_, **m**_6_, **m**_2_} and {Δ_1,2_^1^, Δ_1,3_^2^, Δ_1,5_^3^, Δ_1,4_^4^, Δ_1,6_^5^}, respectively.  Figure 4(b) shows the configuration of** X** where, for sake of easiness, only the two distances Δ_1,2_^1^ and Δ_1,5_^3^ have been represented. Then, the depuration ratio *R* is calculated. This process is an iterative computation that begins with the shortest distance Δ_1,2_^1^. The distance Δ_1,2_^1^ (see Figure 4(c)), corresponding to **z**_1_ and **z**_2_, produces the evaluation of their medium point *u*  ((**z**_1_ + **z**_2_)/2). Since *f*(*u*) is worse than *f*(**z**_1_) but not worse than *f*(**z**_2_), the element **z**_2_ is considered to be part of the same concentration as **z**_1_. The same conclusion is obtained for Δ_1,3_^2^ in case of **z**_3_, after considering the point *v*. For Δ_1,5_^3^, the point *w* is produced. Since *f*(*w*) is worse than *f*(**z**_1_) and *f*(**z**_5_), the element **z**_5_ is considered to be part of the concentration corresponding to the next optimum. The iterative process ends here, after assuming that the same result is produced with Δ_1,4_^4^ and Δ_1,6_^5^, for **z**_4_ and **z**_6_, respectively. Therefore, the depuration ratio *D*_*R*_ is calculated as 85% of the distances Δ_1,5_^3^. Once the elements inside of *D*_*R*_ have been removed from** Z**, the same process is applied to the new** Z**. As a result, the final configuration of the memory is shown in Figure 4(d).

### 3.4. Complete MCS Algorithm

Once the new operators (D) memory mechanism, (E) new selection strategy, and (F) depuration procedure have been defined, the proposed MCS algorithm can be summarized by Algorithm 3. The new algorithm combines operators defined in the original CS with the new ones. Despite these new operators, the MCS maintains the same three adjustable parameters (*p*_*a*_, *N*, and gen) compared to the original CS method.

## 4. Experimental Results

This section presents the performance of the proposed algorithm beginning from Section 4.1 that describes the experimental methodology. For the sake of clarity, results are divided into two sections, Section 4.2 and Section 4.3, which report the comparison between the MCS experimental results and the outcomes produced by other multimodal metaheuristic algorithms.

### 4.1. Experimental Methodology

This section examines the performance of the proposed MCS by using a test suite of fourteen benchmark functions with different complexities.  Table 3 in the appendix presents the benchmark functions used in our experimental study. In the table,** NO** indicates the number of optimal points in the function and *S* indicates the search space (subset of *R* ^2^). The experimental suite contains some representative, complicated, and multimodal functions with several local optima. Such functions are considered complex entities to be optimized, as they are particularly challenging to the applicability and efficiency of multimodal metaheuristic algorithms. A detailed description of each function is given in the appendix.

In the study, five performance indexes are compared: the effective peak number (EPN), the maximum peak ratio (MPR), the peak accuracy (PA), the distance accuracy (DA), and the number of function evaluations (NFE). The first four indexes assess the accuracy of the solution, whereas the last measures the computational cost.

The effective peak number (EPN) expresses the amount of detected peaks. An optimum **o**_*j*_ is considered as detected if the distance between the identified solution **z**_*j*_ and the optimum **o**_*j*_ is less than 0.01 (||**o**_*j*_ − **z**_*j*_|| < 0.01). The maximum peak ratio (MPR) is used to evaluate the quality and the number of identified optima. It is defined as follows:

(15) $$\begin{array}{c} \text{MPR}=\frac{\sum_{i=1}^{t}f\left(z_{i}\right)}{\sum_{j=1}^{q}f\left(o_{j}\right)}, \end{array}$$

where *t* represents the number of identified solutions (identified optima) for the algorithm under testing and *q* represesnts the number of true optima contained in the function. The peak accuracy (PA) specifies the total error produced between the identified solutions and the true optima. Therefore, PA is calculated as follows:

(16) $$\begin{array}{c} PA=\overset{q}{\underset{j=1}{\sum }}\left|f\left(o_{j}\right)-f\left(z_{j}\right)\right|. \end{array}$$

Peak accuracy (PA) may lead to erroneous results, mainly if the peaks are close to each other or hold an identical height. Under such circumstances, the distance accuracy (DA) is used to avoid such error. DA is computed as PA, but fitness values are replaced by the Euclidian distance. DA is thus defined by the following model:

(17) $$\begin{array}{c} \text{DA}=\overset{q}{\underset{j=1}{\sum }}||o_{j}-z_{j}||. \end{array}$$

The number of function evaluations (NFE) indicates the total number of function computations that have been calculated by the algorithm under testing, through the overall optimization process.

The experiments compare the performance of MCS against the crowding differential evolution (CDE) [22], the fitness sharing differential evolution (SDE) [21, 22], the clearing procedure (CP) [26], the elitist-population strategy (AEGA) [28], the clonal selection algorithm (CSA) [30], and the artificial immune network (AiNet) [31].

Since the approach solves real-valued multimodal functions and a fair comparison must be assured, we have used for the GA approaches a consistent real coding variable representation and uniform operators for crossover and mutation. The crossover probability of *P*_*c*_ = 0.8 and the mutation probability of *P*_*m*_ = 0.1 have been used. We have employed the standard tournament selection operator with tournament size = 2 for implementing the sequential fitness sharing, the clearing procedure, and the elitist-population strategy (AEGA). On the other hand, the parameter values for the AiNet algorithm have been defined as suggested in [31], with the mutation strength of *β* = 100, the suppression threshold of *σ*_*s*(aiNet)_ = 0.2, and the update rate of *d* = 40%. Algorithms based on DE use a scaling factor of *F* = 0.5 and a crossover probability of *P*_*c*_ = 0.9. The crowding DE employs a crowding factor of CF = 50 and the sharing DE considers *α* = 1.0 with a share radius of *σ*_share_ = 0.1.

In case of the MCS algorithm, the parameters are set to *p*_*a*_ = 0.25, the population size is *N* = 50, and the number of generations is gen = 500. Once they have been all experimentally determined, they are kept for all the test functions through all experiments.

To avoid relating the optimization results to the choice of a particular initial population and to conduct fair comparisons, we perform each test 50 times, starting from various randomly selected points in the search domain as it is commonly done in the literature.

All algorithms have been tested in MatLAB© over the same Dell Optiplex GX520 computer with a Pentium-4 2.66G-HZ processor, running Windows XP operating system over 1 Gb of memory. The sections below present experimental results for multimodal optimization problems which have been divided into two groups. The first one considers functions *f*_1_–*f*_7_, while the second gathers functions *f*_8_–*f*_14_.

### 4.2. Comparing MCS Performance for Functions *f*_1_–*f*_7_

This section presents a performance comparison for different algorithms solving the multimodal problems *f*_1_–*f*_7_ that are shown in Table 3. The aim is to determine whether MCS is more efficient and effective than other existing algorithms for finding all multiple optima of *f*_1_–*f*_7_. All the algorithms employ a population size of 50 individuals using 500 successive generations.

Table 1 provides a summarized performance comparison among several algorithms in terms of the effective peak number (EPN), the maximum peak ratio (MPR), the peak accuracy (PA), the distance accuracy (DA), and the number of function evaluations (NFE). The results are averaged by considering 50 different executions.

Considering the EPN index, in all functions *f*_1_–*f*_7_, MCS always finds better or equally optimal solutions. Analyzing results of function *f*_1_, the CDE, AEGA, and the MCS algorithms reach all optima. In case of function *f*_2_, only CSA and AiNet have not been able to detect all the optima values each time. Considering function *f*_3_, only MCS can detect all optima at each run. In case of function *f*_4_, most of the algorithms detect only half of the total optima but MCS can recognize most of them. Analyzing results of function *f*_5_, CDE, CP, CSA, and AiNet present a similar performance whereas SDE, AEGA, and MCS obtain the best EPN values. In case of *f*_6_, almost all algorithms present a similar performance; however, only the CDE, CP, and MCS algorithms have been able to detect all optima. Considering function *f*_7_, the MCS algorithm is able to detect most of the optima whereas the rest of the methods reach different performance levels.

By analyzing the MPR index in Table 1, MCS has reached the best performance for all the multimodal problems. On the other hand, the rest of the algorithms present different accuracy levels, with CDE and SDE being the most consistent.

Considering thePA index, MCS presents the best performance. Since PA evaluates the accumulative differences of fitness values, it could drastically change when one or several peaks are not detected (function *f*_3_) or when the function under testing presents peaks with high values (function *f*_4_). For the case of the DA index in Table 1, it is evident that the MCS algorithm presents the best performance providing the shortest distances among the detected optima.

Analyzing the NFE measure in Table 1, it is clear that CSA and AiNet need fewer function evaluations than other algorithms considering the same termination criterion. This fact is explained by considering that both algorithms do not implement any additional process in order to detect multiple optima. On the other hand, the MCS method maintains a slightly higher number of function evaluations than CSA and AiNet due to the inclusion of the depuration procedure. The rest of the algorithms present a considerable higher NFE value.

It can be easily deduced from such results that the MCS algorithm is able to produce better search locations (i.e., a better compromise between exploration and exploitation) in a more efficient and effective way than other multimodal search strategies by using an acceptable number of function evaluations.

### 4.3. Comparing MCS Performance for Functions *f*_8_–*f*_14_

This section presents a performance comparison for different algorithms solving the multimodal problems *f*_8_–*f*_14_ that are shown in Table 3. The aim is to determine whether MCS is more efficient and effective than its competitors for finding multiple optima in *f*_8_–*f*_14_. All the algorithms employ a population size of 50 individuals using 500 successive generations.  Table 2 provides a summarized performance comparison among several algorithms in terms of the effective peak number (EPN), the maximum peak ratio (MPR), the peak accuracy (PA), the distance accuracy (DA), and the number of function evaluations (NFE). The results are averaged by considering 50 different executions.

The goal of multimodal optimizers is to find as many as possible global optima and good local optima. The main objective in these experiments is to determine whether MCS is able to find not only optima with prominent fitness value, but also optima with low fitness values.  Table 2 provides a summary of the performance comparison among the different algorithms.

Considering the EPN measure, it is observed that MCS finds more optimal solutions for the multimodal problems *f*_8_–*f*_14_ than the other methods. Analyzing function *f*_8_, only MCS can detect all optima whereas CP, AEGA, CSA, and AiNet exhibit the worst EPN performance.

Functions *f*_9_–*f*_12_ represent a set of special cases which contain a few prominent optima (with good fitness value). However, such functions present also several optima with bad fitness values. In these functions, MCS is able to detect the highest number of optimum points. On the contrary, the rest of algorithms can find only prominent optima.

For function *f*_13_, four algorithms (CDE, SDE, CP, and MCS) can recognize all optima for each execution. In case of function *f*_14_, numerous optima are featured with different fitness values. However, MCS still can detect most of the optima.

In terms of number of the maximum peak ratios (MPR), MCS has obtained the best score for all the multimodal problems. On the other hand, the rest of the algorithms present different accuracy levels.

A close inspection of Table 2 also reveals that the proposed MCS approach is able to achieve the smallest PA and DA values in comparison to all other methods.

Similar conclusions to those in Section 4.2 can be established regarding the number of function evaluations (NFE). All results demonstrate that MCS achieves the overall best balance in comparison to other algorithms, in terms of both the detection accuracy and the number of function evaluations.

## 5. Conclusions

The cuckoo search (CS) algorithm has been recently presented as a new heuristic algorithm with good results in real-valued optimization problems. In CS, individuals emulate eggs (contained in nests) which interact in a biological system by using evolutionary operations based on the breeding behavior of some cuckoo species. One of the most powerful features of CS is the use of Lévy flights to generate new candidate solutions. Under this approach, candidate solutions are modified by employing many small changes and occasionally large jumps. As a result, CS can substantially improve the relationship between exploration and exploitation, still enhancing its search capabilities. Despite such characteristics, the CS method still fails to provide multiple solutions in a single execution. In order to overcome such inconvenience, this paper proposes a new multimodal optimization algorithm called the multimodal cuckoo search (MCS). Under MCS, the original CS is enhanced with multimodal capacities by means of (1) incorporation of a memory mechanism to efficiently register potential local optima according to their fitness value and the distance to other potential solutions, (2) modification of the original CS individual selection strategy to accelerate the detection process of new local minima, and (3) inclusion of a depuration procedure to cyclically eliminate duplicated memory elements.

MCS has been experimentally evaluated over a test suite of the fourteen benchmark multimodal functions. The performance of MCS has been compared to some other existing algorithms including the crowding differential evolution (CDE) [22], the fitness sharing differential evolution (SDE) [21, 22], the clearing procedure (CP) [26], the elitist-population strategy (AEGA) [28], the clonal selection algorithm (CSA) [30], and the artificial immune network (AiNet) [31]. All experiments have demonstrated that MCS generally outperforms all other multimodal metaheuristic algorithms in terms of both the detection accuracy and the number of function evaluations. The remarkable performance of MCS is explained by two different features: (i) operators (such as Lévy flight) allow a better exploration of the search space, increasing the capacity to find multiple optima, and (ii) the diversity of solutions contained in the memory** M** in the context of multimodal optimization is maintained and further improved through an efficient mechanism.

## Figures and Tables

**Algorithm 1:**
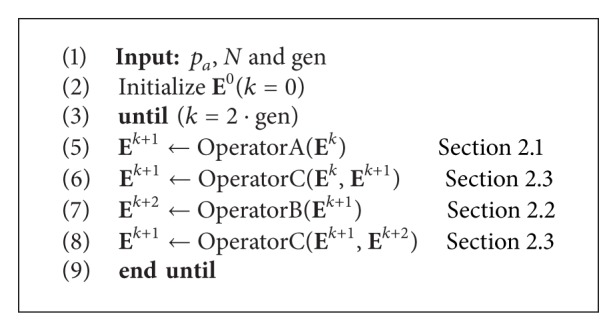
Cuckoo search (CS) algorithm.

**Algorithm 2:**
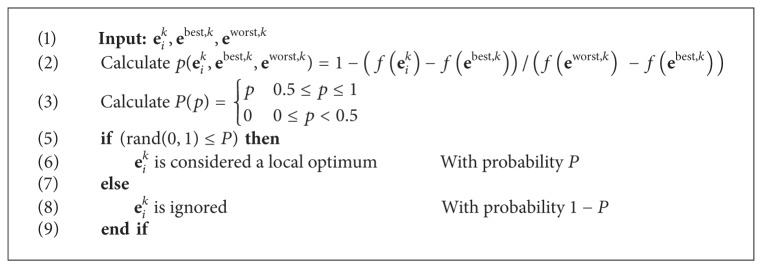
Nonsignificant fitness value rule procedure.

**Procedure 1:**
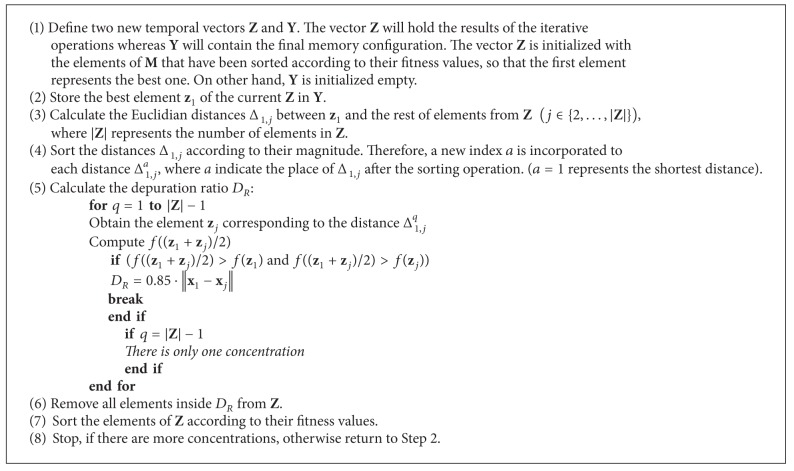


**Algorithm 3:**
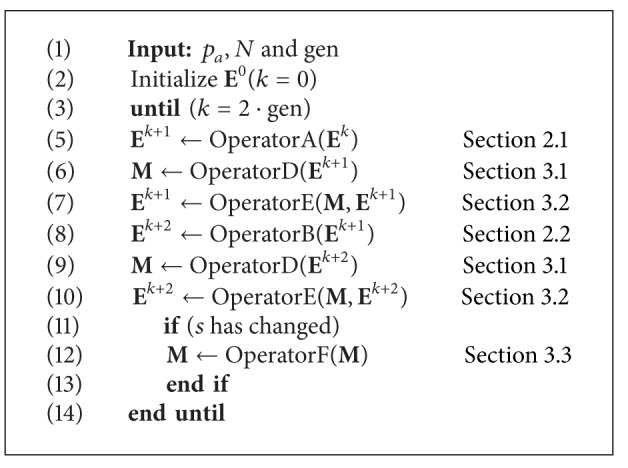
Multimodal cuckoo search (MCS) algorithm.

**Table 1:** Performance comparison among multimodal optimization algorithms for the test functions *f*_1_–*f*_7_. For all the parameters, numbers in parentheses are the standard deviations.

Function	Algorithm	EPN	MPR	PA	DA	NFE
*f*_1_	CDE	3 (0)	0.9996 (0.0004)	0.0995 (0.1343)	0.0305 (0.0169)	27432 (1432)
	SDE	2.96 (0.18)	0.9863 (0.0523)	1.3053 (0.8843)	0.1343 (0.0483)	31435 (2342)
	CP	2.93 (0.25)	0.9725 (0.0894)	1.3776 (1.0120)	0.1432 (0.0445)	34267 (4345)
	AEGA	3 (0)	0.9932 (0.0054)	0.0991 (0.2133)	0.1031 (0.0065)	30323 (2316)
	CSA	2.91 (0.20)	0.9127 (0.0587)	1.4211 (1.0624)	0.2188 (0.0072)	25050 (0)
	AiNet	2.94 (0.20)	0.9002 (0.0901)	1.3839 (1.0214)	0.1760 (0.0067)	25050 (0)
	MCS	3 (0)	1 (0)	0.0005 (0.0001)	0.0007 (0.0002)	25433 (54)

*f*_2_	CDE	12 (0)	1 (0)	0.0015 (0.0010)	0.2993 (0.0804)	26321 (1934)
	SDE	12 (0)	1 (0)	0.0018 (0.0006)	0.3883 (0.0657)	32563 (1453)
	CP	12 (0)	1 (0)	0.0009 (0.0003)	0.2694 (0.0506)	30324 (3521)
	AEGA	12 (0)	0.9987 (0.0011)	0.0988 (0.0097)	0.3225 (0.0058)	29954 (1987)
	CSA	11.92 (0.41)	0.9011 (0.0091)	0.1055 (0.0121)	0.4257 (0.0096)	25050 (0)
	AiNet	11.96 (0.30)	0.9256 (0.0074)	0.0996 (0.0105)	0.3239 (0.0081)	25050 (0)
	MCS	12 (0)	1 (0)	0.0001 (0.0001)	0.0073 (0.0002)	25188 (42)

*f*_3_	CDE	23.03 (1.77)	0.8780 (0.0956)	180.47 (265.54)	9.3611 (6.4667)	28654 (2050)
	SDE	20.06 (2.59)	0.6980 (0.1552)	155.52 (184.59)	14.892 (7.5935)	31432 (1017)
	CP	21.03 (1.90)	0.7586 (0.1125)	192.32 (146.21)	11.195 (3.1490)	32843 (2070)
	AEGA	20.45 (1.21)	0.7128 (0.1493)	134.47 (157.37)	16.176 (8.0751)	30965 (2154)
	CSA	18.02 (2.41)	0.5875 (0.1641)	185.64 (104.24)	21.057 (10.105)	25050 (0)
	AiNet	19.24 (2.01)	0.6123 (0.1247)	179.35 (164.37)	18.180 (9.1112)	25050 (0)
	MCS	24.66 (1.01)	0.9634 (0.0397)	2.9408 (4.3888)	15.521 (8.0834)	25211 (37)

*f*_4_	CDE	3.46 (1.00)	0.4929 (0.1419)	395.46 (305.01)	210.940 (72.99)	29473 (3021)
	SDE	3.73 (0.86)	0.5301 (0.1268)	544.48 (124.11)	206.65 (160.84)	33421 (1342)
	CP	3.26 (0.63)	0.4622 (0.0869)	192.32 (146.21)	199.41 (68.434)	29342 (1543)
	AEGA	3.51 (0.52)	0.5031 (0.0754)	188.23 (101.54)	187.21 (33.211)	32756 (1759)
	CSA	3.12 (0.11)	0.4187 (0.0464)	257.54 (157.18)	278.14 (47.120)	25050 (0)
	AiNet	3.20 (0.47)	0.5164 (0.0357)	197.24 (86.21)	178.23 (29.191)	25050 (0)
	MCS	6.26 (0.82)	0.8919 (0.1214)	41.864 (16.63)	39.938 (12.962)	25361 (81)

*f*_5_	CDE	22.96 (2.25)	0.4953 (0.0496)	0.2348 (0.0269)	17.83 (7.1214)	28543 (1345)
	SDE	31.40 (2.35)	0.6775 (0.0503)	0.7005 (0.0849)	3.9430 (0.9270)	30543 (1576)
	CP	21.33 (2.00)	0.4599 (0.0436)	1.3189 (0.5179)	10.766 (1.9245)	28743 (2001)
	AEGA	30.11 (2.01)	0.6557 (0.0127)	0.8674 (0.0296)	2.870 (1.6781)	29765 (1911)
	CSA	24.79 (3.14)	0.5107 (0.0308)	0.2121 (0.0187)	8.7451 (3.470)	25050 (0)
	AiNet	26.57 (2.35)	0.5005 (0.0471)	0.2087 (0.0324)	6.472 (2.4187)	25050 (0)
	MCS	33.03 (2.07)	0.8535 (0.0251)	0.1617 (0.0283)	4.6012 (1.4206)	25159 (49)

*f*_6_	CDE	6 (0)	0.9786 (0.0157)	0.1280 (0.0942)	0.1231 (0.0182)	30234 (2410)
	SDE	5.86 (0.43)	0.9185 (0.0685)	0.3842 (0.1049)	0.1701 (0.0222)	31453 (1154)
	CP	6 (0)	0.9423 (0.0123)	0.3460 (0.0741)	0.1633 (0.0149)	30231 (832)
	AEGA	5.11 (0.64)	0.8945 (0.0387)	0.4004 (0.0879)	0.1224 (0.0101)	31932 (943)
	CSA	4.97 (0.24)	0.8174 (0.0631)	0.4797 (0.0257)	0.1295 (0.0054)	25050 (0)
	AiNet	5.23 (1)	0.9012 (0.0197)	0.3974 (0.0702)	0.1197 (0.0054)	25050 (0)
	MCS	6 (0)	0.9993 (0.0002)	0.0037 (0.0014)	0.0006 (0.0002)	25463 (37)

*f*_7_	CDE	30.36 (2.77)	0.6200 (0.0566)	2.2053 (1.8321)	330.51 (47.531)	33423 (1021)
	SDE	35.06 (5.15)	0.7162 (0.1051)	1.9537 (0.9290)	243.39 (140.04)	32832 (995)
	CP	35.06 (3.98)	0.7164 (0.0812)	2.4810 (1.4355)	250.11 (78.194)	31923 (834)
	AEGA	32.51 (2.59)	0.7004 (0.0692)	2.0751 (0.9561)	278.78 (46.225)	33821 (1032)
	CSA	31.78 (1.14)	0.6764 (0.4100)	1.9408 (0.9471)	347.21 (38.147)	25050 (0)
	AiNet	34.42 (1.80)	0.7237 (0.0257)	1.8632 (0.0754)	261.27 (61.217)	25050 (0)
	MCS	38.86 (1.54)	0.8014 (0.0313)	0.2290 (0.0166)	49.53 (7.1533)	25643 (97)

**Table 2:** Performance comparison among multimodal optimization algorithms for the test functions *f*_8_–*f*_14_. For all the parameters, numbers in parentheses are the standard deviations.

Function	Algorithm	EPN	MPR	PA	DA	NFE
*f*_8_	CDE	24.16 (2.77)	0.9682 (0.0318)	2.4873 (2.4891)	0.8291 (0.8296)	28453 (2345)
	SDE	18.56 (2.51)	0.4655 (0.0636)	30.21 (43.132)	2.1162 (0.6697)	31328 (945)
	CP	8.80 (1.95)	0.2222 (0.0509)	60.52 (56.056)	6.9411 (0.9500)	30743 (1032)
	AEGA	15.67 (2.21)	0.3934 (0.0534)	40.56 (10.111)	3.2132 (0.2313)	32045 (684)
	CSA	14.54 (3.12)	0.3323 (0.0431)	48.34 (8.343)	3.8232 (0.4521)	25050 (0)
	AiNet	16.78 (2.63)	0.4264 (0.0321)	37.32 (10.432)	2.9832 (0.5493)	25050 (0)
	MCS	24.73 (0.49)	0.9898 (0.0170)	0.900 (1.4771)	0.2584 (0.1275)	25582 (74)

*f*_9_	CDE	2.1 (0.20)	0.7833 (0.0211)	23.235 (7.348)	2.9354 (0.3521)	30074 (1621)
	SDE	2.3 (0.31)	0.8245 (0.0145)	20.745 (8.012)	2.6731 (0.8621)	31497 (245)
	CP	2.4 (0.25)	0.8753 (0.0301)	18.563 (5.865)	2.3031 (0.7732)	29746 (1114)
	AEGA	2.1 (0.10)	0.7879 (0.0174)	22.349 (6.231)	3.0021 (0.6431)	30986 (1027)
	CSA	2 (0)	0.7098 (0.0025)	32.859 (8.659)	3.1432 (0.5431)	25050 (0)
	AiNet	2 (0)	0.7165 (0.0076)	31.655 (6.087)	3.2265 (0.3467)	25050 (0)
	MCS	4.74 (0.25)	0.9154 (0.0163)	2.3515 (2.511)	0.0109 (0.0428)	26043 (112)

*f*_10_	CDE	4.12 (0.78)	0.7285 (0.0342)	3.546 (1.034)	3.0132 (0.5321)	29597 (1034)
	SDE	4.64 (0.54)	0.7893 (0.0532)	3.054 (1.127)	2.864 (0.3271)	32743 (964)
	CP	4 (0)	0.7092 (0.0298)	3.881 (1.154)	3.3412 (0.4829)	28463 (1142)
	AEGA	3.43 (0.33)	0.6734 (0.0745)	4.212 (1.312)	3.9121 (0.8732)	29172 (1044)
	CSA	3.76 (0.51)	0.6975 (0.0828)	4.002 (1.197)	3.5821 (0.7498)	25050 (0)
	AiNet	4 (0)	0.7085 (0.0385)	3.797 (1.002)	3.3002 (0.6496)	25050 (0)
	MCS	6.82 (0.75)	0.9274 (0.0137)	0.423 (0.064)	0.6842 (0.0598)	25873 (88)

*f*_11_	CDE	10.36 (1.60)	0.8572 (0.1344)	1.859 (0.952)	0.5237 (0.0321)	34156 (2321)
	SDE	10.36 (2.04)	0.8573 (0.1702)	1.268 (0.581)	0.6927 (0.0921)	32132 (975)
	CP	9.16 (1.76)	0.7577 (0.1462)	2.536 (0.890)	0.6550 (0.0440)	30863 (1002)
	AEGA	8.34 (1.32)	0.6954 (0.1021)	4.432 (1.232)	0.7021 (0.0231)	31534 (852)
	CSA	8 (0)	0.6532 (0.1378)	4.892 (1.003)	0.7832 (0.0432)	25050 (0)
	AiNet	8 (0)	0.6438 (0.2172)	4.921 (1.102)	0.7753 (0.0326)	25050 (0)
	MCS	12 (0)	0.9998 (0.0003)	0.011 (0.008)	0.0060 (0.0012)	25789 (121)

*f*_12_	CDE	6.21 (1.54)	0.6986 (0.1893)	4.029 (1.401)	5.1514 (1.0351)	31456 (975)
	SDE	5.34 (2.03)	0.5812 (0.1992)	5.075 (1.071)	6.0117 (1.1517)	32481 (1002)
	CP	6.04 (0.61)	0.6312 (0.1771)	4.657 (1.321)	5.3177 (1.7517)	33123 (563)
	AEGA	4 (0)	0.4112 (0.0343)	6.341 (1.034)	7.8751 (1.652)	32634 (843)
	CSA	4 (0)	0.3998 (0.0212)	6.151 (1.121)	7.7976 (1.0043)	25050 (0)
	AiNet	4 (0)	0.4034 (0.0973)	6.003 (1.735)	7.6613 (1.1219)	25050 (0)
	MCS	9.65 (1.45)	0.9411 (0.0087)	0.015 (0.009)	0.1043 (0.0864)	25832 (65)

*f*_13_	CDE	13 (0)	1 (0)	0.010 (0.003)	0.031 (0.0098)	31572 (962)
	SDE	13 (0)	1 (0)	0.008 (0.004)	0.021 (0.0065)	33435 (1201)
	CP	13 (0)	1 (0)	0.015 (0.002)	0.037 (0.0065)	31834 (799)
	AEGA	10.66 (1.21)	0.8323 (0.0343)	0.088 (0.033)	0.096 (0.0098)	32845 (1182)
	CSA	8.94 (2.34)	0.7998 (0.0564)	0.110 (0.088)	0.113 (0.0104)	25050 (0)
	AiNet	10.32 (1.52)	0.8297 (0.0206)	0.098 (0.075)	0.087 (0.0086)	25050 (0)
	MCS	13 (0)	0.9997 (0.0134)	0.011 (0.007)	0.023 (0.0016)	25740 (101)

*f*_14_	CDE	3.04 (1.34)	0.6675 (0.0754)	0.809 (0.101)	176.54 (21.23)	32273 (1004)
	SDE	3.55 (0.56)	0.7017 (0.0487)	0.675 (0.079)	115.43 (34.21)	30372 (965)
	CP	2.87 (1.23)	0.6123 (0.0861)	1.081 (0.201)	202.65 (42.81)	31534 (1298)
	AEGA	3 (0)	0.6686 (0.0542)	0.894 (0.076)	150.32 (57.31)	29985 (1745)
	CSA	3 (0)	0.6691 (0.0231)	0.897 (0.045)	161.57 (27.92)	25050 (0)
	AiNet	3.50 (0.25)	0.7001 (0.0765)	0.668 (0.097)	121.43 (43.12)	25050 (0)
	MCS	7.13 (0.81)	0.9859 (0.0094)	0.023 (0.010)	17.62 (4.13)	25786 (92)

**Table 3:** Test functions used in the experimental study.

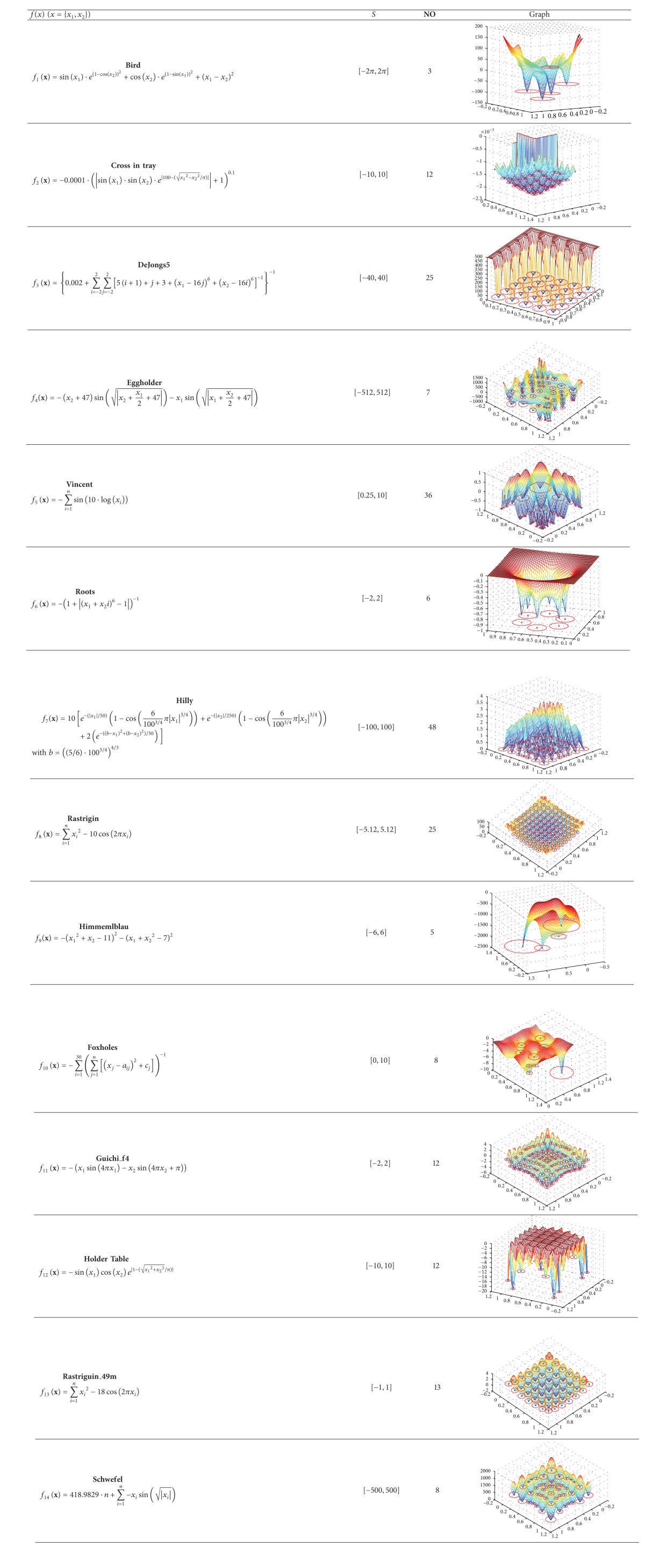

## Appendix

For list of benchmark functions, see Table 3.
